# Data collaboration analysis in predicting diabetes from a small amount of health checkup data

**DOI:** 10.1038/s41598-023-38932-x

**Published:** 2023-07-21

**Authors:** Go Uchitachimoto, Noriyoshi Sukegawa, Masayuki Kojima, Rina Kagawa, Takashi Oyama, Yukihiko Okada, Akira Imakura, Tetsuya Sakurai

**Affiliations:** 1 Master’s Program in Service Engineering, University of Tsukuba, Tsukuba, Japan; 2 Faculty of System and Information Engineering, University of Tsukuba, Tsukuba, Japan; 3 Institute of Medicine, University of Tsukuba, Tsukuba, Japan; 4 Health Department, National Health Insurance Division, Tsukuba, Japan; 5 Center for Artificial Intelligence Research, University of Tsukuba, Tsukuba, Japan; 6 Faculty of Science and Engineering, Hosei University, Tokyo, Japan

**Keywords:** Preventive medicine, Information technology

## Abstract

Recent studies showed that machine learning models such as gradient-boosting decision tree (GBDT) can predict diabetes with high accuracy from big data. In this study, we asked whether highly accurate prediction of diabetes is possible even from small data by expanding the amount of data through data collaboration (DC) analysis, a modern framework for integrating and analyzing data accumulated at multiple institutions while ensuring confidentiality. To this end, we focused on data from two institutions: health checkup data of 1502 citizens accumulated in Tsukuba City and health history data of 1399 patients collected at the University of Tsukuba Hospital. When using only the health checkup data, the ROC-AUC and Recall for logistic regression (LR) were 0.858 ± 0.014 and 0.970 ± 0.019, respectively, while those for GBDT were 0.856 ± 0.014 and 0.983 ± 0.016, respectively. When using also the health history data through DC analysis, these values for LR improved to 0.875 ± 0.013 and 0.993 ± 0.009, respectively, while those for GBDT deteriorated because of the low compatibility with a method used for confidential data sharing (although DC analysis brought improvements). Even in a situation where health checkup data of only 324 citizens are available, the ROC-AUC and Recall for LR were 0.767 ± 0.025 and 0.867 ± 0.04, respectively, thanks to DC analysis, indicating an 11% and 12% improvement. Thus, we concluded that the answer to the above question was “Yes” for LR but “No” for GBDT for the data set tested in this study.

## Introduction

The number of patients with type 2 diabetes mellitus (“diabetes”) is increasing worldwide^1,2^ . Worsening diabetes is known to increase the likelihood of various serious complications such as retinopathy, which can cause blindness, and nephropathy, which is the leading cause of kidney failure, neuropathy, and macrovascular disease and directly or indirectly causes death^3^. Therefore, it is essential to predict and prevent diabetes onset. Lifestyle interventions alone can significantly reduce the risk of developing diabetes to the same extent as pharmacological interventions if they can be predicted early^4^.

Various factors help predict the risk of developing diabetes, including obesity, smoking, and a family history of diabetes^4^. However, it is known that the risk of developing diabetes varies by country and ethnicity^5^; China^6^, Germany^7^, Thailand^8^, Japan^9^, and various other countries have developed their models. Traditional models use classical statistical models, such as logistic regression and Cox proportional hazards, which have been criticized for their inability to describe nonlinear relationships among covariates^10–12^. However, in recent years, large amounts of data have become available, and models have been actively developed using state-of-the-art machine learning methods^13,14^. In Japan, there is an initiative called the “Data Health Plan” that analyzes data accumulated from health checkups conducted by prefectural and municipal governments to predict and prevent lifestyle-related diseases such as diabetes and metabolic syndrome at an early stage^15^. Using diagnostic data from Osaka Prefecture, Seto et al. predicted whether a person would develop diabetes within 3 years using gradient-boosting decision tree (GBDT) and logistic regression (LR). The results showed that GBDT was more reliable than LR when the amount of data was sufficient. In addition, Ooka et al. used health examination data from Yamanashi Prefecture to predict whether HbA1c would increase within 1 year^16^. The availability of large datasets has been a critical factor for the success of these studies. This is especially true in areas where the population is declining and aging and compact cities are being developed.

In situations where it is difficult for individual institutions, such as municipalities and hospitals, to collect large datasets, a framework that integrates data accumulated at multiple institutions into a single large-scale data set while maintaining confidentiality and returns the results obtained by analyzing the integrated single large-scale data to each institution has recently attracted attention, especially in the field of machine learning. Such frameworks have recently received particular attention, especially in machine learning. Such frameworks are collectively referred to as distributed data analysis. Federated learning is a typical distributed data analysis method^17^. Although this method has been tested for its effectiveness in various applications, the need to use the same model at each institution and communication costs have been cited as challenges. An alternative approach to address these challenges is data collaboration (DC) analysis proposed by Imakura and Sakurai^18^. Bogdanova et al. compared the two methods and experimentally demonstrated that DC analysis is more valuable than federated learning when the number of institutions is small^19^. In addition, DC analysis has the advantage of lower communication costs than federated learning, which is advantageous for the distributed data analysis of medical data from multiple institutions, as envisioned in this study.

Based on the above background, we asked whether highly accurate prediction of diabetes is possible even from small data by expanding the amount of data through DC analysis. The aim of this study is to answer this question experimentally.

## Data and methodology

This study is part of the “Research and Development of Next Generation Artificial Intelligence Technology for Improving Productivity through Data Collaboration Analysis” in the “Realization of Smart Society by Applying Artificial Intelligence Technology” project by the New Energy and Industrial Technology Development Organization (NEDO). The handling of medical data was approved by the Ethical Review Committee for Clinical Research of the University of Tsukuba Hospital (permission number: H30-187).

### Data

The data used were the specified health examination data of the National Health Insurance insured in Tsukuba City from 2014 to 2018 (hereafter referred to as “health checkup data”) and the examination history data of the University of Tsukuba Hospital (hereafter referred to as “health history data”). Tsukuba City specified health checkup data from health checkups taken by the National Health Insurance insured persons in Tsukuba City. Approximately 4000 data items were available for tests performed at the hospital. The health checkups are examinations for people aged 40–74 years, and the checkups focus on metabolic syndrome to prevent lifestyle-related diseases. Data items included the results of the health checkup, such as data on the patient’s height, weight, and fasting plasma glucose (FPG) level, as well as data from a questionnaire that confirmed the health history of stroke, heart disease, and other diseases and items related to usual lifestyle habits, such as diet and smoking. The data used are from 2014 to 2018, and the data for each year are for all citizens who received a health checkup in Tsukuba City in that year. The data used in this experiment are the data of all citizens who received the specified health checkups conducted by Tsukuba City each year, and the data of 116 items exist. The data for the University of Tsukuba Hospital health history were those of patients with a history of nephrology examination at the University of Tsukuba Hospital and whose FBG level or hemoglobin A1c (HbA1c) level were measured. The common feature values between the two data sets are listed in Table 1.

**Table 1:** Features that are commonly available in the two data sets.

Attribute	Unit
Age	Years-old
Sex	Qualitative: (Male) or (Female)
Hemoglobin A1c (HbA1c)	%
Hemoglobin content (Hb)	g/dl
Fasting plasma glucose (FPG)	mg/dl
Casual blood glucose (CBG)	mg/dl
Red blood cell count (RBC)	10${}^4$/m${}^3$
Urinary glucose (UG)	Qualitative: (−), (±), (+), (++), or (+++)
Urinary protein (UP)	Qualitative: (−), (±), (+), (++), or (+++)
High density lipoprotein-cholesterol (HDL-C)	mg/dl
Low density lipoprotein-chilesterol (LDL-C)	mg/dl
Aspartate aminotransferase (AST)	U/1
$\gamma$-glutamyltransferase ($\gamma$-GT)	U/1
Serum creatinine (Scr)	mg/dl
Hematcrit (Hct)	%

### Data cleaning

The formats of the health checkup and health history data were first aligned. In the health checkup data, each characteristic of each year for each patient is the value observed during one health checkup per year. In contrast, in the health checkup data, because a patient may have multiple checkups in a year, the mean value of the observed values from multiple checkups was used for each characteristic for each year for each patient for the quantitative data and the mode value for the qualitative data. For urinary glucose and urine protein, measurements were assigned as (−), (±), (+), (++), or (+++). In this experiment, the observation period was 2 years, 2014 and 2015. In what follows, we use samples to mean citizens or patients. To make predictions from 2 years of data, we extracted data on at least one feature in both 2014 and 2015. The result was 7,920 samples for the health checkup data and 15,666 for the health history data. We then identified samples as having both HbA1c and FPG levels at least once from 2016 to 2018 as diabetic if they met the Japanese clinical practice guideline^20^ at least once and not diabetic otherwise; those with both HbA1c and FPG at least once during these 3 years were extracted. The results of this study are for the health checkup data. This resulted in 1399 samples in the health checkup data and 1531 in the health history data. Subsequently, features with missing values of 50% or more were removed. Because the features to be deleted are different in each dataset, FPG and urinary glucose are deleted in the health checkup data. The missing values of a qualitative (quantitative) variable were imputed by the mean (mode) value of the feature. Since there are no missing values in the objective labels by the above construction, the inputs to the models including LR and GBDT are the same.

**Figure 1:**
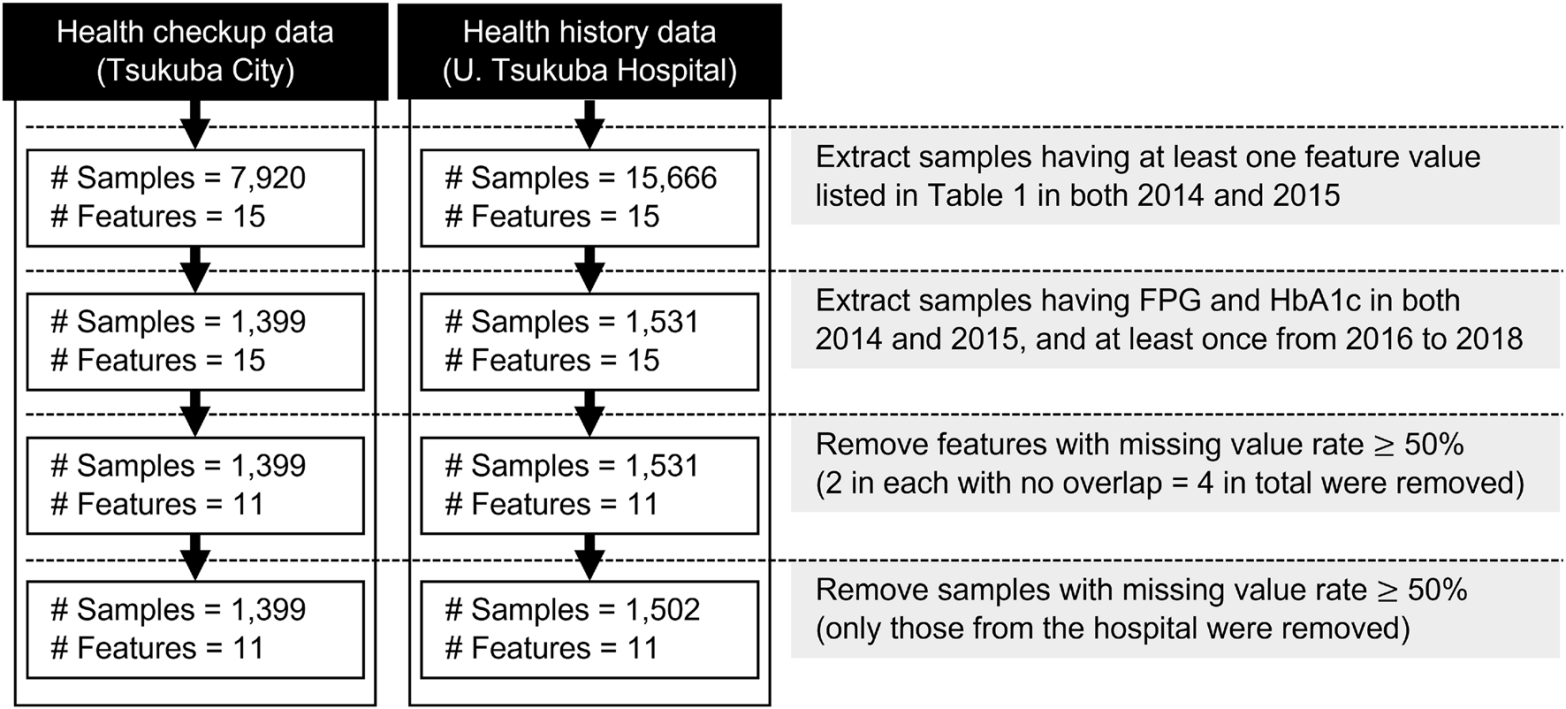
Flow chart of our pre-processing for the data set where *N* indicates the number of samples.

Finally, patients with fewer than half of all features were excluded. The final number of samples was 1399 for the Tsukuba City health checkup data and 1502 for health history data. A data preprocessing flow diagram summarizing the above is shown in Fig. 1.

### Method

All procedures involving human participants were in accordance with the ethical standards of the 1964 Helsinki Declaration and its later amendments. The need for informed consent was waived by the Ethics Committee of University of Tsukuba Hospital (IRB Approval Number H30-187) because all data were anonymized according to the Japanese Ethical Guidelines for Medical and Health Research Involving Human Subjects enacted by the Ministry of Health, Labor, and Welfare of Japan (https://www.mhlw.go.jp/content/10600000/000757206.pdf (in Japanese)).

The performance of the two types of analysis methods was compared. The first is an individual analysis, namely, a conventional analysis. This analysis used only the 1399 samples of the health checkup data obtained by the above-mentioned preprocessing. Ninety-nine samples were randomly selected as test data, and the remaining 1300 were used as training data. One hundred experiments were conducted to evaluate the prediction performance using confidence intervals. The second is DC analysis. This analysis added the 1502 samples of the health history data to training data through data collaboration analysis. However, the test data were the same as before, that is, 99 randomly selected cases from the health checkup data. The analysis aimed to experimentally observe the difference in prediction performance between individual and DC analyses. Therefore, the number of samples from the health history data was not varied. Furthermore, several experiments were conducted varying amounts of samples. These experiments aimed to verify the effectiveness of DC analysis, assuming a municipality smaller than Tsukuba City. The data set in which all 1,300 training data of the health checkup data described above are used is called “All,” the data set in which half of them are used is called “Half,” and the data set in which another half of them are used is called “Quarter.” To ensure fairness, the same test data were used for the three data sets.

As the prediction models, logistic regression (LR) and gradient-boosting decision tree (GBDT) were used. LightGBM was used to implement GBDTs. Also, $\ell _1$ regularization was used for the regularization term in LR. Decision trees, random forest, and XGBoost were also compared in the preliminary experiments. However, the performance of these three methods was not as good as that of LR and GBDTs; therefore, the details of their performance will not be discussed further.

### Outcome

The objective variable was a binary label indicating whether a sample (i.e., a citizen) was diagnosed with diabetes (at least once) in 2016, 2017, and 2018. The onset of diabetes was detected according to the diagnostic criteria presented in the guidelines for diabetes care^20^, which are based on HbA1c and FPG levels. Specifically, an index of HbA1c of $\ge$ 6.5% and FPG level of $\ge$ 126 mg/dl were used.

### Evaluation indicators and models

One hundred bootstraps were used to evaluate predictive performance with the confidence intervals indicated. The ROC-AUC (hereafter referred to as “AUC”) was used as the evaluation index. Because the data handled in this experiment were highly unbalanced, the recall, precision, and their harmonic mean known as F1-score (F-measure) were also used as evaluation indices, unlike in previous studies.

### Data collaboration (DC) analysis

DC analysis was proposed by Imakura and Sakurai^18^ as a method for integrative analysis of multiple distributed data sets. Unlike Federated Learning proposed by Konečný et al.^17^, DC analysis does not share models but rather shares intermediate representation data transformed using dimensionality reduction methods such as principal component analysis (PCA)^21^. The following is a brief description of the actual algorithm. The actual algorithm is briefly described below.

DC analysis works in two roles: worker and master. Let *n* be the number of workers in the collaboration and assume that each worker has data ${X_i\in R}^{m_i\times d} (1\le i\le n)$, where $m_i$ is the number of samples held by the worker and *d* is the number of dimensions of data held by the worker. First, the data $X_i$ held by each worker is transformed into intermediate representation data ${\widetilde{X}}_i$ using a dimensionality compression method $f_i$ such as principal component analysis PCA as follows:

$$\begin{aligned} {\widetilde{X}}_i=f_i\left( X_i\right) \in R^{m_i\times l_i} \end{aligned}$$

At this time, the number of dimensions of the intermediate representation obtained satisfies $d>l_i$. The generated intermediate representation data cannot be integrated with data from other workers for analysis because the number of dimensions differs for each worker. Therefore, we introduce anchor data $X^{anc}\in \mathbb {R}^{r\times d}$, common to all workers. For the generation of anchor data, Imakura and Sakurai^18^ use a random number matrix, etc., while Imakura et al.^22^ use the synthetic minority over-sampling technique (often referred to as SMOTE for short). The anchor data is then converted to an intermediate representation using the dimensionality compression method $f_i$ when each worker generates the intermediate representation,

$$\begin{aligned} {{\widetilde{X}}_i}^{anc}=f_i\left( X^{anc}\right) \in \mathbb {R}^{r\times l_i} \end{aligned}$$

Workers share ${\widetilde{X}}_i$ and ${{\widetilde{X}}_i}^{anc}$ with the master. The master obtains the collaborative representation ${\hat{X}}_i$ by re-projecting the intermediate representation data ${\widetilde{X}}_i$ using the reconversion function $g_i$ such that ${\hat{X}}_i=g_i\left( {\widetilde{X}}_i\right) \in R^{m_i\times k}$. The reconversion function $g_i$ is then effectively determined by the solution of the perturbation minimization problem targeting

$$\begin{aligned} {{\hat{X}}_i}^{anc}\approx {{\hat{X}}_j}^{anc}, \quad \ {{\hat{X}}_i}^{anc}=g_i\left( {{\widetilde{X}}_i}^{anc}\right) , \end{aligned}$$

Now, *k* is the number of dimensions after integration common to all periods and can be freely determined. Finally, the master combines the collaborative representation ${\hat{X}}_i$ in the sample direction and analyzes it as a single data set.

## Results

### Participant characteristics

**Table 2:** Basic statistics of the features adopted in this study where *N* means the number of samples and SS stands for statistical significance (***if $p\le 0.001$, ** if $p\le 0.01$, and * if $p\le 0.05$).

	Health checkup data			Health history data		
	Positive	Negative	SS	Positive	Negative	SS
	(*N* = 43)	(*N* = 1356)		(*N* = 209)	(*N* = 1293)	
Age	66.5 (3.8)	64.5 (6.8)		65.0 (13.5)	58.8 (18.8)	***
HbA1c	6.4 (0.4)	5.8 (0.4)	***	7.0 (0.9)	6.2 (0.8)	***
Hb	14.5 (1.1)	12.0 (1.1)	***	12.9 (1.9)	13.0 (1.7)	
RBC	476.9 (34.2)	457.9 (34.9)	***	424.9 (62.3)	431.2 (56.8)	
HDL-C	57.8 (14.4)	63.7 (15.3)	*	49.3 (11.8)	51.1 (10.7)	*
LDL-C	117.7 (25.0)	121.5 (26.8)		101.5 (24.5)	105.6 (22.1)	*
AST	24.3 (6.4)	23.4 (5.6)		28.7 (15.0)	27.6 (14.4)	
$\gamma$-GT	34.9 (23.2)	29.3 (19.3)		44.2 (35.5)	40.7 (36.2)	
Hct	43.6 (2.6)	42.1 (2.9)	***	38.4 (5.1)	38.8 (4.6)	
Sex	M: 27, F: 16	M: 706, F: 650		M: 126, F: 83	M: 693, F: 600	
UP	40, 3, 0, 0, 0	1279, 77, 0, 0, 0		166, 12, 9, 11, 11	1160, 22, 34, 56, 21	***

**Figure 2:**
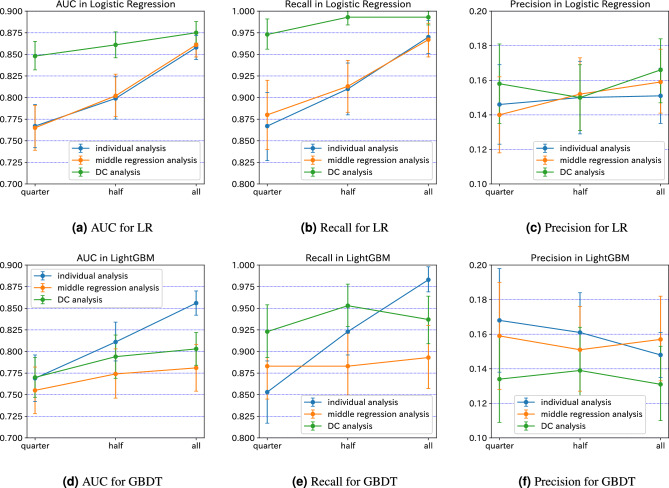
AUC (left), Recall (middle), and Precision (right) for LR (top) and GBDT (bottom) in the three data sets, i.e., “quarter”, “half”, and “all”.

The 11 features adopted in this study through the aforementioned preprocessing and their statistics for the 2015 data are described in Table 2 where the sample mean and standard deviation are shown in the form of “mean (std)” for each qualitative data, and the frequency distribution is shown for each qualitative data. The difference between the two data sets in terms of the mean values of these 11 features were all statistically significant ($p\le 0.001$) except for one feature, age. The results of the similar comparisons between the positive and negative samples in each data set are shown in the table. The point was that FPG and HbA1c levels were used to define the positive labels following the guidelines for diabetes care^20^ while they were not adopted as the features due to their high missing rate in the data set. The positive group in the health checkup data had higher values for AST, Hct, HbA1c, $\gamma$-GT, Hb, and RBC than the opposing group. The “−” percentage of “±” was higher than that of “−” for UP. The positive group also included a higher percentage of male patients. The positive group had higher AST, HbA1c, and $\gamma$-GT levels and a lower percentage of “−” UP than the opposing group.

### Model performance

The results for LR and GBDT are summarized in Fig. 2. There are three analysis results: individual (raw data) analysis, middle regression analysis, and DC analysis. Here, middle regression analysis is a method in which raw data $X_i$ is analyzed by converting $\tilde{X}_i$ through dimensionality reduction by PCA. Figure 2 shows that for the “All” data set when LR was used, DC analysis was superior to the individual (raw data) analysis and the intermediate representation analysis for all four evaluation indicators in terms of mean values. However, when using GBDT, DC analysis was below the individual analysis for all metrics. Next, we turn to the “Half” data set, which assumes a small municipality. Again, when using LR, DC analysis outperformed the individual analysis and the intermediate representation analysis for all metrics. However, when using GBDT, DC analysis was lower than the individual raw data analysis, except for recall. In addition, when looking at the confidence intervals for each evaluation metric, especially for LR, DC analysis was superior to the individual analysis in terms of Recall and AUC. For the ‘Quarter” data set, which assumes even smaller municipalities, DC analysis was also superior to the raw data individual and intermediate representation analyses for all metrics in terms of mean values when using LR. However, when using GBDT, DC analysis was inferior to the individual analysis in terms of Precision. In addition, when looking at the confidence intervals for each of the metrics, especially for LR, DC analysis outperformed the individual analysis in terms of Recall and AUC.

**Table 3:** Increase in the averages of the evaluation metrics of 100 bootstrap trials DC analysis for LR and GBDT where positive values imply improvement while negative values imply deterioration.

Method	Evaluation metric	Data set		
		Quarter	Half	All
(a) Compared to the individual (raw data) analysis				
LR	AUC	0.081 (11%)	0.061 (8%)	0.017 (2%)
	Precision	0.012 (8%)	0.000 (0%)	0.015 (10%)
	Recall	0.107 (12%)	0.083 (9%)	0.023 (2%)
	F1 score	0.029 (13%)	0.008 ($4\%$)	0.021 ($9\%$)
GBDT	AUC	0.001 ($0\%$)	$-0.017$ ($-2\%$)	$-0.053$ ($-6\%$)
	Precision	$-0.034$ ($-20\%$)	$-0.022$ ($-14\%$)	$-0.017$ ($-11\%$)
	Recall	0.070 ($8\%$)	0.030 ($3\%$)	$-0.046$ ($-5\%$)
	F1 score	$-0.044$ ($-17\%$)	$-0.039$ ($-15\%$)	$-0.040$ ($-16\%$)
(b) Compared to the middle regression analysis				
LR	AUC	0.083 ($10\%$)	0.059 ($7\%$)	0.014 ($2\%$)
	Precision	0.018 ($13\%$)	$-0.002$ ($-1\%$)	0.007 ($4\%$)
	Recall	0.093 ($11\%$)	0.080 ($9\%$)	0.026 ($3\%$)
	F1score	0.033 ($15\%$)	0.005 ($2\%$)	0.011 ($4\%$)
GBDT	AUC	0.015 ($2\%$)	0.020 ($3\%$)	0.022 ($3\%$)
	Precision	$-0.025$ ($-16\%$)	$-0.012$ ($-8\%$)	$-0.026$ ($-16\%$)
	Recall	0.040 ($5\%$)	0.070 ($8\%$)	0.044 ($5\%$)
	F1score	$-0.023$ ($-0.1\%$)	$-0.019$ ($-8\%$)	$-0.032$ ($-13\%$)

Table 3, which summarizes the averages of the evaluation metrics for LR, shows that DC analysis improved the values of AUC and Recall to a greater extent than the individual analysis and the intermediate expression individual analysis, in particular, indicating an 11% and 8% improvement for AUC.

## Discussion

In this study, we used DC analysis to analyze the data held by different institutions to build a model for predicting the future of diabetes. Consequently, the prediction accuracy of diabetes was improved by expanding the number of samples through DC analysis for LR but not for GBDT. For LR, the averages were improved using DC analysis for almost all metrics in all datasets when compared to the individual (raw data) analysis as summarized in Table 3a as well as when compared to the middle regression analysis as summarized in Table 3b. In addition, for AUC and Recall in particular, more significant improvement was observed when the data set was “Quarter” or “Half” data set.

However, as mentioned above and seen from Table 3a, GBDT was inferior to the individual (raw data) analysis for most data sets and associated metrics. There are three reasons for this. First, no parameter tuning was performed on the machine learning methods in this experiment. Specifically, there is no limit on the depth, the learning rate is set to 0.1, and the number of estimators is set to 100. More sophisticated parameter tuning, such as limiting the depth of GBDT, is a promising future direction. Second, the combination of GBDT and the data sets used in this study may be incompatible with the intermediate representation method. Recall that the intermediate representation method, PCA in this study, is employed to process raw data for confidential data sharing in DC analysis. Since the middle regression analysis is the counterpart of the individual analysis for the processed data, the performance of the middle regression analysis basically gets worse than the individual analysis (for raw data) as can be seen from Fig. 2. On the other hand, the performance of DC analysis basically gets better than the middle regression analysis thanks to the data sharing as can be seen from Table 3b at least for AUC and Recall even for GBDT. The point is how much the middle regression analysis gets worse than the individual analysis. We see from Fig. 2 that the gap between performances of the individual (raw data) analysis and the middle regression analysis is slight for LR but significant for GBDT. We note that this problem might be specific to the data set used in this study; for example, Imakura et al.^23^ showed that DC analysis improves the performance of decision tree using 12 data sets including artificial one and those from UCI machine learning repository. We are still not sure when the aforementioned gap between the individual (raw data) analysis and the middle regression analysis can get small theoretically nor empirically. However, in practice, what we need to do is to observe the gap in the individual analysis, before getting into DC analysis, by changing the method for intermediate representation to find out the best one. However, promising methods, including SVD (Singular value decomposition) and LPP (Locality preserving projection) as well as PCA, perform all similarly in the preliminary experiment of this study. Searching for effective methods for intermediate representation is a future problem. Third, in terms of sample size, Seto et al. concluded that GBDT showed better AUC than LR when the sample size exceeded 10,000. However, since the largest data set in this experiment is only approximately 1500, GBDT may show a higher AUC than LR if this values increases.

One limitation of this study was the question of labeling. When we built the model to predict diabetes within 3 years, we defined the target patients as those not diagnosed with diabetes at baseline and had FPG and HbA1c levels at least once within 3 years. If the number of patients was limited to those with these two values in all 3 years, the number would be 164 in the city health checkup data and 724 in the hospital health history data, and if we built the model as in this analysis, there would be no data for 2016 and 2018, only for 2017.

## Conclusion

Existing studies have reported that diabetes can be discriminated with high accuracy from a large amount of health checkup data using state-of-the-art machine learning models, such as gradient-boosting decision tree (GBDT). In this study, we asked whether highly accurate prediction of diabetes is possible even from small data by expanding the amount of data through data collaboration (DC) analysis, which is a framework for integrating and analyzing data accumulated at multiple institutions while ensuring confidentiality. To this end, we focused on data from two institutions: health checkup data of 1502 citizens accumulated in Tsukuba City and health history data of 1399 patients collected at the University of Tsukuba Hospital. We found that a high-performance prediction model with ROC-AUC close to 0.9 could be constructed thanks to DC analysis. This is achieved by logistic regression (LR) with L1 regularization, but not by GBDT. Therefore, the answer to the above question was “Yes” for LR but “No” for GBDT for the data set tested in this study.

Although this study targeted the health checkup data of Tsukuba City, some municipalities only collected smaller amounts of health checkup data, such as those treated in the experiment. Even assuming a municipality smaller than Tsukuba City, we found that the accuracy can be improved by expanding the data through data collaboration analysis. This trend is expected to become more pronounced as rural areas become more compact. However, the present results suggest that, even in such cases, a high-performance diabetes prediction model can be constructed using DC analysis, in other words, the high performance of LR might be robust to changes in the sample size. On the other hand, we are not sure how the performance of GBDT as well as LR is sensitive to changes in the data sets or parameters as discussed in the previous section. The University of Tsukuba Hospital, which participated in DC analysis in this study, is a hospital with specific functions in Japan. It plays a leading role in the development of medical technology and is expected to contribute to the improvement of medical care in depopulated areas in the form of the setting treated in this study. In addition, this study did not improve the accuracy of GBDT, a decision tree-based method. Therefore, future studies should investigate why nonlinear methods such as GBDT lose accuracy in DC analysis.

## Data Availability

The data that support the findings of this study are available from Tsukuba city and University of Tsukuba Hospital but restrictions apply to the availability of these data, which were used under license for the current study, and so are not publicly available. Data are however available from the corresponding author upon reasonable request and with permission of Tsukuba city and University of Tsukuba Hospital.

## References

[1] Charvat H (2015). Impact of population aging on trends in diabetes prevalence: A meta-regression analysis of 160,000 Japanese adults. J. Diabetes Invest..

[2] Cho NH (2018). Idf diabetes atlas: Global estimates of diabetes prevalence for 2017 and projections for 2045. Diabetes Res. Clin. Pract..

[3] Fowler MJ (2008). Microvascular and macrovascular complications of diabetes. Clin. Diabetes.

[4] Zimmet P, Alberti K, Shaw J (2001). Global and societal implications of the diabetes epidemic. Nature.

[5] Ramachandran A, Ma RCW, Snehalatha C (2010). Diabetes in Asia. Lancet.

[6] Zhou X (2013). Nonlaboratory-based risk assessment algorithm for undiagnosed type 2 diabetes developed on a nation-wide diabetes survey. Diabetes Care.

[7] Schulze MB (2007). An accurate risk score based on anthropometric, dietary, and lifestyle factors to predict the development of type 2 diabetes. Diabetes Care.

[8] Aekplakorn W (2006). A risk score for predicting incident diabetes in the thai population. Diabetes Care.

[9] Nanri A (2015). Development of risk score for predicting 3-year incidence of type 2 diabetes: Japan epidemiology collaboration on occupational health study. PLoS ONE.

[10] Noble D, Mathur R, Dent T, Meads C, Greenhalgh T (2011). Risk models and scores for type 2 diabetes: Systematic review. Bmj.

[11] Collins GS, Mallett S, Omar O, Yu L-M (2011). Developing risk prediction models for type 2 diabetes: A systematic review of methodology and reporting. BMC Med..

[12] Obermeyer Z, Emanuel EJ (2016). Predicting the future-big data, machine learning, and clinical medicine. N. Engl. J. Med..

[13] Tuppad A, Patil SD (2022). Machine learning for diabetes clinical decision support: A review. Adv. Comput. Intell..

[14] Kodama S (2022). Predictive ability of current machine learning algorithms for type 2 diabetes mellitus: A meta-analysis. J. Diabetes Investig..

[15] Seto H (2022). Gradient boosting decision tree becomes more reliable than logistic regression in predicting probability for diabetes with big data. Sci. Rep..

[16] Ooka T (2021). Random forest approach for determining risk prediction and predictive factors of type 2 diabetes: Large-scale health check-up data in japan. BMJ Nutr. Prevent. Health.

[17] Konečnỳ, J. *et al.* Federated learning: Strategies for improving communication efficiency. arXiv preprint arXiv:1610.05492 (2016).

[18] Imakura A, Sakurai T (2020). Data collaboration analysis framework using centralization of individual intermediate representations for distributed data sets. ASCE-ASME J. Risk Uncertain. Eng. Syst. Part A Civ. Eng..

[19] Bogdanova, A., Nakai, A., Okada, Y., Imakura, A. & Sakurai, T. Federated learning system without model sharing through integration of dimensional reduced data representations. arXiv preprint arXiv:2011.06803 (2020).

[20] Araki E (2020). Japanese clinical practice guideline for diabetes 2019. Diabetol. Int..

[21] Pearson KL (1901). on lines and planes of closest fit to systems of points in space. London Edinburgh Dublin Philosoph. Mag. J. Sci..

[22] Imakura A, Kihira M, Okada Y, Sakurai T (2023). Another use of smote for interpretable data collaboration analysis. Expert Syst. Appl..

[23] Imakura A, Inaba H, Okada Y, Sakurai T (2021). Interpretable collaborative data analysis on distributed data. Expert Syst. Appl..

